# Parenting interventions for incarcerated mothers

**DOI:** 10.3389/fpsyt.2025.1658663

**Published:** 2025-11-21

**Authors:** Alexis Clark, Leah Lancellotta, Tom D. Kennedy

**Affiliations:** College of Psychology, Nova Southeastern University, Fort Lauderdale, FL, United States

**Keywords:** maternal incarceration, family dynamics, family relationships, parenting interventions, systematic review

## Abstract

As women constitute the fastest-growing prison population, understanding the effects of incarceration is essential. This poses a significant interest in a subset of women who are also mothers. The mental health challenges faced by incarcerated women are profound and unique compared to those of their male counterparts. Likewise, the mental health challenges of mothers may be viewed as separate and distinct. Thus, these factors may impact the relationships between women and their families. This systematic review was conducted using the PRISMA framework. It examines parenting interventions designed for justice-involved women, particularly incarcerated mothers, and how these programs affect maternal well-being, parenting skills, and family relationships. The electronic databases PsycINFO and PubMed were searched for the systematic review. In phase one, 3,720 articles were extracted. Of these, 1,572 articles were screened for title and abstract irrelevancy. Following screening, 1,209 were removed due to title irrelevance, and 347 were removed for abstract irrelevance. After removing any studies that were not intervention-based, a total of 15 full-text articles remained for screening. In this phase, the articles were reviewed for quality, and six were excluded. Justice-involved mothers face unique challenges that require comprehensive and tailored interventions. These challenges may be best addressed through trauma-informed, family-oriented programming and mental health treatment.

**Systematic review registration:**

https://www.crd.york.ac.uk/prospero/, identifier CRD420251031672.

## 1 Introduction

Correctional and forensic psychology has grown considerably in terms of its wellness services, building a strong foundation for evidence-based practices. In doing so, the existing literature has brought awareness to the unique challenges and mental health outcomes of incarcerated individuals. Historically, most of the research and clinical applications in this field have focused predominantly on male populations. However, women currently constitute the fastest-growing population in prison. Most incarcerated women are mothers of minor children whose caregiving arrangements are often disrupted (1, 2). In many cases, the child’s father is absent prior to the mother’s incarceration, leaving grandparents, foster families, or other relatives to assume caregiving responsibilities (3). Thus, children of incarcerated mothers often experience multiple caregiver transitions and visitation and contact are less consistent, creating significant emotional distress for mothers who identify with their parental roles (4). This gendered caregiving dynamic highlights a key distinction between incarcerated men and women. When fathers are incarcerated, the children’s mother generally continues her caretaking role, preserving a sense of stability for the child. However, incarcerated mothers attempt to “mother from prison” by maintaining emotional connections and caregiving identities, despite separation and institutional barriers (1, 4, 5). This dual reality—rising incarceration rates for women and their simultaneous efforts to sustain parenting—creates a unique set of challenges that extend beyond individual mental health to affect children, families, and communities.

Recent data from the Bureau of Justice Statistics indicate that in 2023, there were 85,900 women serving sentences of more than one year in state or federal prisons, a 4% increase from 82,500 in 2022 (6). In local jails, the female population reached 95,100 in 2023, up from 92,900 in 2022 (7). Despite some decline in total incarceration rates, the number of women incarcerated in jails and prisons has increased substantially over the past decade (6–8). While the female incarcerated population has continued to rise, research and interventions have largely remained centered on men, leaving a gap in understanding and addressing the distinct challenges faced by women. Over the past 20 years, researchers have shown a growing interest in areas such as risk assessment, mental health services, and interventions tailored for male offenders. A significant gap exists in the literature of evidence-based practices developed explicitly for women.

Incarcerated women represent a vulnerable population, bearing a longstanding history of trauma, mental illness, and disrupted social ties. However, embedded within this group is an even more distinct and frequently marginalized subgroup: incarcerated mothers. While women in general are more likely than men to experience PTSD, depression, and substance use disorders (9, 10), mothers in prison face the compounded challenge of managing these conditions alongside the psychological burden of separation from their children. This intersection — being both female and a mother in custody — creates unique vulnerabilities that are not fully captured when incarcerated women are studied as a homogeneous group. Mental health issues are pervasive among incarcerated women, with approximately 76% reporting past or current mental health problems and over half having a substance use disorder in the year prior to incarceration. Earlier data also show that about 19% of incarcerated women experienced serious psychological distress in the 30 days preceding incarceration (2). A significant body of research has highlighted the presence of trauma and psychological distress in female offenders, mainly due to sexual victimization and subsequent mental health conditions. Moreover, these histories are disproportionately prevalent among female offenders (10). Although many incarcerated women report histories of trauma, mental health concerns, and sexual violence, incarcerated individuals in general often undergo intake screenings where severe psychiatric conditions remain undetected. Trestman and colleagues (9) found that a substantial proportion of inmates not initially identified as acutely mentally ill were later shown to have current or lifetime psychiatric disorders. Therefore, it is recognized that many individuals with significant mental health needs may pass through the system without receiving appropriate treatment or support. This is particularly problematic for incarcerated mothers, whose challenges may not be as visible or externalized, leaving them with little to no support.

Embedded within the broader population of female offenders is a subset of mothers who comprise a unique and frequently marginalized group in the context of mental health care. As of 2023, approximately 190,600 women and girls were incarcerated across state, federal, local, juvenile, and immigration detention facilities in the United States. Among women in state prisons, about 58% are mothers of minor children, which often results in significant family disruption (2). Additional research suggests that about 75% of incarcerated women are mothers and primary caregivers (1). Many continue their maternal roles while in prison, experiencing significant stress that negatively affects their physical and mental health (1). However, not all incarcerated mothers maintain contact with their children. Some lose custody before imprisonment due to involvement with child protective services, while others permanently lose parental rights during incarceration. Under the Adoption and Safe Families Act (11), mothers serving longer sentences or lacking kinship care arrangements face a heightened risk of having their children placed in foster care or adopted. In some cases, women give birth while incarcerated and may have their infants permanently removed shortly after delivery (5). These realities underscore that motherhood within correctional settings adds layers of complexity beyond those typically encountered by incarcerated women. Just as women offenders present distinct needs and challenges that require more than a mere adaptation of practices designed for men, justice-involved mothers face even greater unique circumstances that also require comprehensive and tailored interventions. Addressing these differences is essential for the effective delivery of tailored interventions in correctional and forensic mental health settings. As the justice system begins to recognize the needs of families with incarcerated mothers, the lack of specific, evidence-based guidance becomes even more pronounced. A mother’s ability to parent and maintain relationships with their family is closely tied to their mental and emotional well-being. Thus, a disruption in this responsibility acts as a psychological burden, strongly linked to emotional consequences such as depression, loneliness, hopelessness, and suicidality (12). These complex needs shape not only the experience of incarceration but also profoundly affect a woman’s relationship with her family.

Despite the barriers of their incarceration, many women express strong desires and often take deliberate actions to mother from behind bars. In doing so, they are not only demonstrating resilience but also investing emotionally in their family’s lives. Systemic structures within carceral institutions often fail to support this aspect of identity and caregiving (5). Separation from family members during one’s incarceration intensifies the psychological strain that many incarcerated mothers face. Therefore, coping with the loss of daily contact often results in profound emotional distress, including guilt, anxiety, and identity disruption (1). While some women develop coping mechanisms such as spiritual practices or mental reframing, others struggle to mitigate the trauma from their disrupted maternal roles. This is especially challenging, as separation contributes to their sense of self-worth and purpose in life. Given this, the emotional weight of disrupted maternal roles is a threat to many mothers’ well-being. It can also interfere with their ability to maintain meaningful parent-child connections both during and after their incarceration (13). These findings point to another critical gap in current interventions, underscoring that incarcerated mothers require targeted, trauma-informed, family-focused programming that strengthens both their psychological health and their ability to sustain familial bonds. Within correctional settings, laying the groundwork for more effective practices will necessitate addressing these dual needs.

These complex realities can be better understood through Attachment Theory, first introduced by Bowlby (1969/14) and later expanded by Ainsworth and colleagues (15). This theory emphasizes the central role of secure, consistent, and emotionally responsive caregiving within a child’s development. A disruption of these bonds, through prolonged separation or inconsistent contact, can put children at greater risk for insecure attachment, psychological distress, and relationship difficulties. These principles are particularly relevant in the context of maternal incarceration, where separation not only impacts the child but also undermines the mother’s ability to fulfill their parental role. Grounding maternal incarceration within the Attachment Theory framework underscores the importance of interventions that preserve parent–child contact, support maternal caregiving capacities, and promote secure attachment despite the barriers imposed by correctional environments.

To address gaps in the existing literature and inform future intervention strategies, this systematic review seeks to answer the following research questions: Among incarcerated mothers (Population), what is the impact of parenting interventions, delivered during incarceration (Intervention), on maternal well-being, parenting skills, and family connection outcomes (Outcomes), based on pre- and post-intervention changes or, where applicable, comparison groups (Comparators)?

Guided by the PICOS framework described by Higgins et al. (16), this review highlights a unique and rapidly growing prison population, consisting of incarcerated mothers. It also includes children, families, and caregivers affected by maternal incarceration. The reviewed articles examine maternal incarceration as the primary exposure and thereby evaluate various treatment methods aimed at addressing its effects. Where applicable, studies include pre/post-intervention assessments or comparison groups to evaluate program impact (Comparators). Outcomes of interest include measurable or observed changes in parenting skills and behaviors, parent–child communication and attachment, caregiver collaboration, maternal mental health, and family connection outcomes. Eligible studies employ a robust methodological framework—either qualitative, quantitative, or mixed methods—and explicitly link their results to the identified intervention strategies (Study designs).

## 2 Methods

A scoping search was conducted to identify any existing reviews or meta-analyses on the effects of maternal incarceration, specifically through a developed intervention. The scoping search identified no existing reviews in this area. Next, a specific protocol was developed and registered with PROSPERO (CRD420251031672) to avoid duplication and ambiguity. Following registration with PROSPERO, the electronic databases of PsycINFO and PubMed were scoured. The Preferred Reporting Items for Systematic Reviews and Meta-Analyses (PRISMA) guidelines were followed in conducting the current review. The initial database search terms included a combination of the following terms: mothers, jail, prison, maternal incarceration, family, and relationships. The results yielded many themes reflective of maternal incarceration; thus, the review was narrowed to include intervention-based studies. References yielded from the search were eliminated if they were duplicates or irrelevant by title or abstract. Of the remaining references, several were removed upon a thorough, in-depth quality evaluation. A modified checklist was adapted from one employed in systematic reviews by Forman-Dolan et al. (17) and Magram et al. (18) to construct the inclusion and exclusion criteria for individual studies. This was formulated from the comparator and outcome questions of the PICO model, ensuring that full-text studies were relevant to the specific population, interventions, and outcomes of interest.

Regarding the composition and provenance of the checklist, the instrument represents a refined adaptation of validated assessment tools from prior systematic reviews in the field, incorporating targeted modifications for incarcerated mothers and parenting-based interventions that were not fully captured by existing checklists (17, 18). These adaptations emphasized parent–child relationship outcomes and intervention specificity, which were central to this review. Iterative pilot testing across 15 studies demonstrated high internal consistency (Cronbach’s α > 0.90) and supported the checklist’s reliability and applicability to our study population, thereby mitigating potential biases in the original frameworks. To further strengthen rigor and reproducibility, the final checklist was applied independently by two reviewers, with discrepancies resolved through consensus, ensuring consistent appraisal across studies.

### 2.1 Inclusion and exclusion criteria

Inclusion criteria were met if

1. The study assessed the impact of maternal incarceration on at least one of the following factors: mental health, physical health, social activities, cognitive functioning, education, risky behaviors, attachment, or support.
2. If it examined the relationship between maternal incarceration and family outcomes or dynamics.
3. If a sound methodological framework was present (e.g., robustness, validity, and reliability of the methodological framework used in the study), with results of the study being directly linked to the aim of the study.
4. If the study identified an intervention strategy related to the impact of maternal incarceration.

Exclusion criteria included the following factors: (a) the participants in the samples were not incarcerated mothers or the participants were not incarcerated in a locked facility (i.e., jail, prison, detention center) in the United States or related territories, (b) no qualitative or quantitative outcomes related to the impact of maternal incarceration were described, (c) the type of article was non-peer reviewed, a book review, editorial, or master thesis and (d) if there was a risk of bias (i.e., selection, performance, detection, attrition or reporting bias). Due to narrowing our scope, we modified our inclusion criteria to include the presence of an intervention. The inclusion and exclusion criteria utilized for appraising the quality of articles are provided in **Table 1**.

**Table 1:** Quality appraisal checklist.

Inclusion criteria
Yes
• The study is focused on incarcerated mothers and explicitly evaluates family-related outcomes through intervention. (one of the following must be checked “yes”)
□ Parenting Skills or Behaviors (measured or observed)
□ Parent-Child Attachment or Bonding (measured or observed)
□ Family Reunification Outcomes (measured or observed)
□ Emotional or Mental Health Impact on the Family Unit (measured or observed)
□ Communication or Relationship Quality (measured or observed)
□ Intergenerational Impact (e.g., effects on children) (measured or observed)
□ Other
• Includes a clear and replicable intervention protocol (methods and procedures)
• Evidence of methodological rigor (e.g., control group, pre-post design, validated measures)
• Were the results directly linked to the aim of the study
• Description of intervention strategies used for improving effects of maternal incarceration
□ Identifies type/modality of intervention
□ Describes where and how often the intervention occurred
□ Outlines specific content or activities involved
□ Mentions any theoretical framework
□ Includes information on facilitators
□ Indicates whether children were involved
□ Notes any cultural tailoring

Exclusion criteria
Yes
Sample population (any of the following are grounds for exclusion)
□ Sample does not consist of currently or formerly incarcerated mothers
□ Participants are not involved in justice system or lack family caregiving roles
□ No outcomes (the following is grounds for exclusion)
□ No discussion of family functioning, relationships, or parenting
Type of article (any of the following are grounds for exclusion)
□ Non-peer-reviewed article
□ Editorial
□ Book review
□ Master’s thesis or dissertation (unless peer-reviewed or published)
Assessing risk of bias (any of the following are grounds for exclusion)
□ Selection bias
□ Performance bias
□ Detection bias
□ Attrition bias
□ Reporting bias

Notably, this review focused exclusively on U.S. populations due to the unique legal, correctional, and treatment contexts within the United States that shape the experiences of incarcerated mothers and their families. In other words, each country has distinct legal policies and social services that shape the experience of maternal incarceration. Moreover, countries around the world vary in how they structure incarceration, with differences in visitation policies, access to interventions, and the benefits offered to incarcerated individuals and their families. Including international populations would not only introduce substantial variability in legal frameworks and correctional practices, but it would also complicate comparison among interventions, limiting the specificity of the conclusions.

## 3 Results

**Figure 1:**
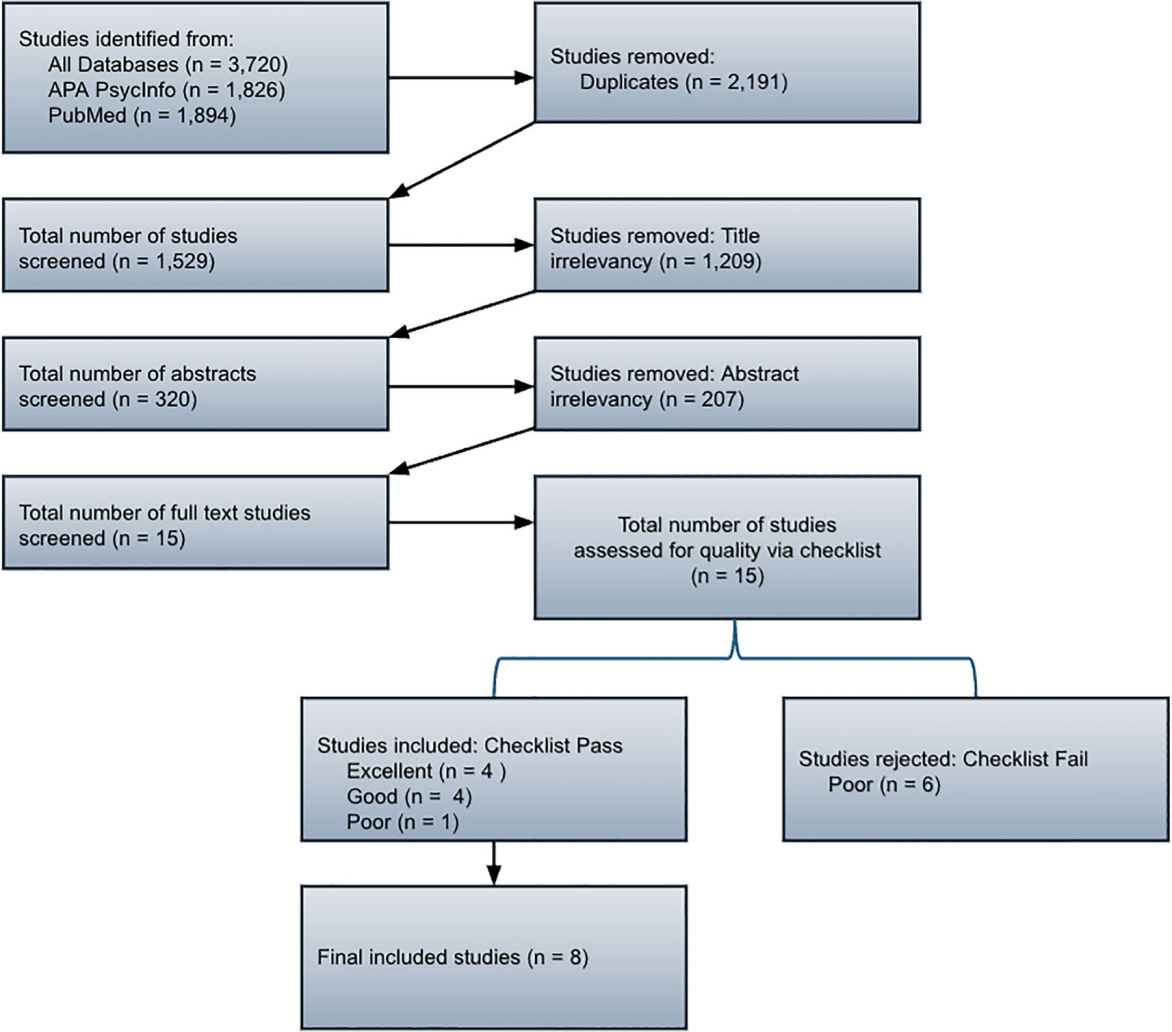
PRISMA flow chart.

The systematic review comprised two electronic databases: PsycINFO and PubMed. The search generated 3720 articles, and 1529 articles were identified after duplicates were removed. Of these, 1209 were removed due to title irrelevancy, and 207 were removed for abstract irrelevancy. This resulted in 113 articles, which were organized by themes, and the scoping review updated the inclusion criteria to include intervention-based studies. This yielded a total of 15 full-text articles. These 15 articles were reviewed for quality, and of these, six were excluded due to concerns about their quality. **Figure 1** outlines the PRISMA inclusion and exclusion process for this systematic review.

### 3.1 Methodological quality

We reviewed the impact of maternal incarceration on various domains of family relationships. Two reviewers independently assessed the methodological quality of 15 full-text articles. Each study was rated using a three-point scale based on methodological rigor and satisfaction of inclusion criteria: 0 = Poor, 1 = Good, and 2 = Excellent. Studies were rated as “Excellent” if they used pre-/post-data along with a comparison group, “Good” if they used pre-/post-data without a comparison group, and “Poor” if they lacked statistical analyses or failed to provide quantitative data. Initial ratings were compared and discussed in consensus meetings to resolve discrepancies. Inter-rater reliability statistics (e.g., kappa coefficient) were not calculated due to the small number of included studies and the qualitative nature of the review. Therefore, the consensus approach allowed for structured discussion and agreement on final inclusion and scoring decisions. A third reviewer was not needed, as all disagreements were resolved through discussion. Of the 15 full-text articles assessed, four studies met all eligibility criteria and were rated as “Excellent,” four were rated as “Good,” and one was rated as “Poor.” Guided by the methodological quality criteria outlined in **Table 2**, eight studies were ultimately included in the review.

**Table 2:** Quality assessment chart.

Quality of the study	Studies evaluated	Relevance	Sample population	Measures and outcomes	Inferential statistics
Excellent	(19) (20) (21) (22)	Used a parenting intervention with a control or comparison group	Mothers incarcerated in correctional facilities	Effects of the intervention were measured using clearly defined, valid, and reliable instruments	Outcomes were described using a pre-/post-design and a comparison group to demonstrate the effects of the intervention
Good	(23) (24) (25) (26)	Used a parenting intervention	Mothers incarcerated in correctional facilities	Effects of the intervention were measured using clearly defined, valid, and reliable instruments	Outcomes were described using a pre-/post-design to demonstrate the effects of the intervention
Poor	(27)	Used a parenting intervention	Mothers incarcerated in correctional facilities	Effects of the intervention were measured through qualitative data gathered through semi-structured interviews	Outcomes were described using themes and participant quotes

While only a small number of studies met full inclusion criteria, this itself reflects a critical gap in the existing literature. The limited pool of eligible studies underscores the urgent need for more targeted, high-quality interventions specifically designed for incarcerated mothers and their families. Despite widespread recognition of the challenges faced by this population, few programs have been rigorously evaluated using robust designs that capture family-level outcomes. This suggests that maternal incarceration remains an under-addressed area, especially with respect to interventions that promote emotional regulation and long-term reunification outcomes.

### 3.2 Characteristics of included studies

All the studies reviewed were conducted in the United States. Most of the samples used in these studies were derived from a diverse set of individuals between 2000 and 2020. For the studies reviewed, the samples included incarcerated mothers who participated in parenting-related interventions while incarcerated. These interventions were conducted within a single facility, and studies did not incorporate participants from multiple incarceration facilities. Although the studies varied in sample size, issues with participant retention during the intervention were a common barrier to success. The ages of mothers and children varied across interventions. A recurring theme across the studies is that many incarcerated mothers have a history of family incarceration, trauma, or systemic involvement, often linked to cycles of poverty, neglect, and substance use. Several interventions aim not only to support current parenting but to interrupt intergenerational cycles of justice involvement. Additionally, many of the mothers across studies were unmarried and incarcerated for drug-related or affiliated charges, reflecting broader patterns of substance use. The sample sizes, participant demographics, type of intervention used, specific measures and instruments employed, and identified outcomes in each article are outlined in **Table 3**.

**Table 3:** Characteristics of study sample.

References	Sample	Race/Ethnicity	Intervention type	Measures/Instruments	Outcome relevant to Intervention
(23)	10 mothers from the Westchester County Department of Corrections (WCDOC), New York State	40% African American, 10% Latina, 10% biracial	Parenting Inside Out (PIO)	Interviews, the DASS-21 Scale, and the Rosenberg Self-Esteem Scale	Some statistically significant results were reported with a small sample size
(24)	10 mothers from the Metropolitan Correctional Center, Federal Jail in Manhattan, New York	50% Latina, 20% African American	Parenting Inside Out (PIO)	Interviews, DASS-21, Guttman Self-Esteem Scale	Some statistically significant results were reported with a small sample size
(19)	106 mothers from an unspecified institution	White (51), African American (43), Other (11)	Parenting From Inside: Making the Mother-Child Connection	PSI-M, PAM, BSI, MOM-OK usage,	Yielded significant results with moderate to large effect sizes
(25)	45 Jailed mothers, unspecified location	26% African American, 63% White	Parenting While Incarcerated curriculum	Adult Adolescent Parenting Inventory, SFP survey, process notes, participant feedback	Yielded significant results with small effect sizes
(21)	161 incarcerated mothers in Kentucky Correctional Institution for Women	58% White, 36% Black, 6% Other minority group	Rebonding and Rebuilding (A Parenting Curriculum)	Class Member Information Questionnaire, AAPI-2, PCRI ​​	Yielded significant results without reporting effect sizes
(20)	71 mothers at a state correctional facility in the Appalachian Region	93% White, non-Hispanic, 3.7% African American, 3.7% Biracial	PCIT	DPICS-III, AAPI-2, PSI, CAP, TAI	Yielded statistically significant results with moderate to large effect sizes
(22)	47 mothers at the Oregon State Correctional Facility	Not reported	Emotions: Taking Care of Yourself and Your Child When You Go Home	Interview, DERS, ATQ, Maternal Emotional Style Questionnaire, Center for Epidemiologic Studies Depression Scale, BSI, SRD	Yielded statistically significant results with moderate to large effect sizes
(26)	104 mothers at the Arkansas Department of Corrections.	46.1% White, 43.1% African American, 6.9% Hispanic, 3.9% American Indian	Parenting from Prison	ISE, AAPI, personal history questionnaire, follow-up questionnaire	Yielded significant results without reporting effect sizes

### 3.3 Review of interventions

Although research discusses interventions for parents, there is limited research that demonstrates the efficacy of parenting interventions within incarcerated populations, specifically mothers. The articles rated as ‘excellent’ represent studies that included a comparison or control group and used a pre-/post-test design. These studies evaluated various parenting interventions, outlined in **Table 4**, for incarcerated mothers, including *Parenting From Inside: Making the Mother-Child Connection*, *Parent-Child Interaction Therapy, The Rebonding and Rebuilding (A Parenting Curriculum)*, and *Emotions: Taking Care of Yourself and Your Child When You Go Home*. The remaining articles identify interventions such as *Parenting Inside Out, Parenting While Incarcerated*, and *Parenting from Prison*.

**Table 4:** Characteristics of interventions.

Intervention	Session/Time length	Components
Parenting From Inside: Making the Mother-Child Connection	8, 2-hour sessions	“Taking Care of Feelings,” “Smart Listening,” “Conversations that Connect,” “Communicating with Your Child through Letters,” “Telephone Visits,” “Connecting with Your Child’s Caregiver,” “Talking to Your Child about Your Offense,” “Giving Guidance When Your Children Are in Trouble”
Parent-Child Interaction Therapy (PCIT)	7 sessions, 90-minute weekly classes	PCIT Protocol
The Rebonding and Rebuilding (A Parenting Curriculum)	12 weeks, meeting twice a week for 3-hour sessions	“Family and Child Development,” “Discipline,” “Difficult Topics,” “Personal Growth,” “Child Abuse,” and “Special Lessons for Incarcerated Parents.”
Emotions: Taking Care of Yourself and Your Child When You Go Home	8 weeks, with 15 2-hour sessions	9 sessions of emotion regulation skills, 6 sessions of targeted emotion coaching skills
Parenting Inside Out (PIO)	2 months, with 2-hour classes meeting twice per week, 14 classes total	“Effective Parenting Styles,” “Effective Speaking Skills,” “Effective Listening Skills,” “Effective Problem Solving,” “Child-Centered Play,” “The Child’s Job, and the Parent’s Job,” “Giving Effective Directions and Encouragement,” “Rules, Rewards and Consequences,” “Time Out With Backup Privilege Removal,” “Going Home, You and Your Children,” and “Healthy Adult Relationships.”
Parenting While Incarcerated	12 weeks, with 1-hour classes weekly	“Introduction to PWI: behavior change,” “Addiction,” “What kids can do,” “Behavior rewards,” “Goals and objective,” “Noticing and ignoring,” “Communication I,” “Communication II,” “Alcohol, tobacco, drug, and families,” “Solving behavior problems and giving directions,” “Setting limits II,” and “graduation celebration.”
Parenting from Prison	15 weeks	“Child development,” “Communication,” “Guidance,” and “Self-esteem”

The *Parenting From Inside: Making the Mother-Child Connection* is a program designed for mothers to reduce parenting stress, strengthen their bond with caregivers, enhance communication patterns with their children, and improve their emotional well-being (19). The curriculum begins with basic cognitive-behavioral skills, including specific skill sets, and progresses to applying these skills across various situations and in challenging contexts. The sessions were led by one of three third and fourth-year clinical psychology doctoral students, along with an inmate co-facilitator. The program consisted of eight two-hour sessions. This program directly supports attachment-related goals by helping mothers obtain skills to reduce parenting stress and strengthen emotional bonds disrupted by incarceration, aligning with attachment theory’s emphasis on consistent and sensitive caregiving. The study consisted of the random assignment of participants to an immediate treatment group versus a waitlist control group, which began 11 weeks after the initial group (19).

The *Parent-Child Interaction Therapy* (PCIT) program consisted of seven 90-minute classes (20). The sessions were divided between curriculum protocol and active behavioral rehearsals, also known as role plays. In the parenting role plays, participants rotated between the roles of parent, child, and coder. The instructors provided feedback and coaching skills in class. Participants were asked to practice their skills daily outside of class with another class member or their child, and to monitor their progress on the PCIT homework sheet, which would be reviewed at the next class. The intervention’s emphasis on role plays, feedback sessions, and opportunities to practice skills with children, when applicable, promotes secure, consistent, and emotionally responsive interactions that support healthy parent–child attachment (20).

The *Rebonding and Rebuilding (A Parenting Curriculum)* intervention combines classroom instruction with an interactive component to enhance parenting and relational skills between mothers and their children (21). The program lasts 12 weeks, meeting twice a week for three hours. Incorporated into the parenting classes are worksheets, written exercises to promote self-evaluation, and opportunities to practice new skills. In addition to the parenting classes, an interactive component consisted of extended visitation time. By providing opportunities for mothers to practice emotional and behavioral responsiveness during extended visits, the program operationalizes attachment theory’s emphasis on maintaining consistent, nurturing interactions to strengthen relational security. Participants were randomly assigned to control and comparison groups, which consisted of mothers who had “never enrolled in parenting classes” (21).

The *Emotions: Taking Care of Yourself and Your Child When You Go Home* program aimed to help incarcerated mothers develop skills in emotion regulation and emotion coaching to support both mothers and their children in managing the stress of incarceration and the challenges of reunification after release (22). The intervention encompasses a parent emotion regulation component and an emotion coaching component. This has been identified as an area of need, as parental validation and support in managing children’s negative emotions can foster stronger emotion regulation skills. These skills are an important protective factor linked to lower rates of depression, anxiety, conduct, and adjustment-related disorders—issues often observed in children of incarcerated parents. Grounded in attachment theory, the *Emotions* program emphasizes the caregiver’s emotional availability as a foundation for secure parent–child relationships. By teaching emotion regulation and coaching strategies within a trauma-informed framework, the program supports healing from trauma, reduces the risk of intergenerational transmission of emotional dysregulation, and strengthens mothers’ capacity to respond sensitively to their children’s needs. Enhancing mothers’ emotion regulation and empathy helps promote consistent, emotionally responsive caregiving—core principles of both attachment and trauma-informed approaches. The program consists of 15 two-hour sessions over eight weeks. Differing from other identified interventions, participation required mothers to have completed the *Parenting Inside Out* program previously and to be within six months of release from prison. Participants were placed in intervention and control groups and were assessed before the program, after the program, while still incarcerated, and six months following their release into the community (22).

The *Parenting Inside Out* program was used in two studies conducted by the same primary author (23, 24). While differing in sample population, the intervention remained consistent. The program consisted of 14 classes, offered twice a week for two months, with each class lasting two hours. During each class, the participants would identify a learning goal that was individualized and expanded upon in their homework assignments. In addition to providing programming, a stress management component, certification in CPR, First Aid, and AED were incorporated. The final day of the program included a graduation and reunification day, which allowed program participants to receive a parenting certificate and have physical contact with their children. From an attachment perspective, this reunification experience provides children with an opportunity to reconnect physically and emotionally with their mothers, reinforcing feelings of safety and continuity in the parent–child relationship. *Parenting Inside Out* incorporated a trauma-informed approach by addressing the emotional and behavioral challenges unique to justice-involved parents. Components like emotion regulation, stress management, communication skills, self-reflection, and supporting positive, non-violent parenting strategies were highlighted in the context of trauma (23, 24).

The *Parenting While Incarcerated* curriculum (25) was developed based on the evidence-based parenting intervention, *Strengthening Families Program* (28). The program was implemented using a one-hour weekly session in a group format. After the program, a graduation ceremony was held. Through ongoing reflection and shared experiences, the program encouraged mothers to build emotionally responsive communication with their children, supporting attachment repair and familial bonds during separation. The formatting of this program included a collaborative component where facilitators documented how mothers responded to the material and incorporated areas of interest proposed by the mothers into the group (25).

*Parenting from Prison* (26) is a 15-week program based on the Nurturing Parent curriculum (29). Topics include child development, guidance, communication, and self-esteem. Trained volunteers taught the program. The program was seeking to identify outcomes for self-esteem, parental attitudes, and mother-child relationships during incarceration (26).

### 3.4 Effectiveness of interventions

Mothers who participated in the *Parenting From Inside: Making the Mother-Child Connection* program reported several positive changes (19). They experienced reduced stress related to parenting and demonstrated improvements in their emotional well-being. Many also began writing more letters to their children, reflecting efforts to maintain emotional connection and continuity in the parent–child relationship, which are essential to secure attachment. According to results from the Parenting Stress Index, mothers felt more confident in their parenting and experienced less stress related to visitation, with effects in the medium-to-large range. There was also a noticeable improvement in how mothers viewed their relationship with their children’s caregivers. These findings, measured by the Parenting Alliance Measure (PAM), showed increased confidence in the caregiver and more substantial alignment in parenting goals. This reflects the program’s emphasis on empathy and open communication. Mental health symptoms such as anxiety, depression, and hostility also decreased after the intervention, as measured by the Brief Symptom Inventory. An important part of the program involved helping mothers recognize and manage their emotional reactions using a framework called MOM-OK. This approach played a significant role in many of the improvements, particularly in reducing parenting stress and improving mental health. However, it had less of an effect on symptoms tied to paranoia and sensitivity in relationships. While both groups improved after participating in the program, the immediate treatment group did not show significantly greater improvement than the waitlist group. This raises questions about whether the program itself caused the changes, and the authors suggest that further research is needed to better understand its effects (19).

The *Parent-Child Interaction Therapy* (PCIT) program utilized the Dyadic Parent-Child Interaction Coding System-III (DPICS-III) as a measure of interaction quality between children and their parents (20). Findings from the DPICS-III demonstrated that mothers in the PCIT group engaged in more positive parenting behaviors, displayed less negative attention during child-led interactions, and showed greater responsiveness to their children, reflecting measurable improvements in the quality of parent–child interactions. Additionally, parenting and child-rearing attitudes were assessed using the Adult-Adolescent Parenting Inventory-Second Edition (AAPI-2). Before the program, mothers reported greater acceptance of corporal punishment as an appropriate disciplinary strategy, but after participation, their attitudes shifted toward rejecting physical discipline and endorsing more constructive approaches, alongside improvements in empathy and parenting confidence. As a measure of parenting stress, the program used the Parenting Stress Index, Third Edition (PSI). The Child Abuse Potential Inventory (CAP) was an assessment used to estimate the risk of committing child physical abuse. Finally, the program utilized the Therapy Attitude Inventory (TAI) to evaluate parental satisfaction upon program completion. In addition to observed behavioral changes, mothers indicated greater satisfaction with their parenting and interactions with their children on the Therapy Attitude Inventory, reflecting their own perceptions of improved relational quality after completing PCIT. The PCIT group was compared to mothers who completed a Department of Education-designed facility program. Both groups reported a decrease in parental stress and a similar decrease in risk for child abuse potential. Participants in the PCIT class demonstrated more positive parenting skills, less negative attention in child-led role plays, and a higher level of treatment satisfaction compared to participants in the facility program. Effect sizes were in the moderate-to-large range, highlighting not only statistical but also meaningful change in parenting behaviors compared to mothers in the facility-based program. Participants in the facility-based program did not demonstrate a change in parenting skills upon program completion. However, participants in the facility program increased their knowledge of child development. The gains in positive parenting behaviors and reductions in negative attention observed in the PCIT program align closely with attachment-based principles, as mothers demonstrated greater sensitivity and responsiveness—core components of secure attachment formation. Notably, child development was not a component of the PCIT program (20).

The *Rebonding and Rebuilding* program used the Parent-Child Relationship Inventory (PCRI) and Adult-Adolescent Parenting Inventory (AAPI-2) to evaluate program effectiveness (21). These assessments were administered on the first and last day of the program. The program’s findings suggest that mothers became more aware of age-appropriate expectations for their children and showed a reduced inclination to use physical or corporal punishment as a disciplinary strategy. The pre-program assessments indicated that many mothers held punitive attitudes toward discipline and accepted corporal punishment as appropriate. Following participation, AAPI-2 results showed a clear shift away from support for corporal punishment, with mothers endorsing more constructive, non-violent strategies and demonstrating greater empathy for their children. Mothers also expressed greater empathy for their children’s feelings and placed more value on their children’s needs over their own. Mothers also reported improvements in the quality of their parent–child relationships, as measured by the PCRI, noting better communication, greater involvement, and stronger perceived support following program completion. These findings indicate that interventions emphasizing emotionally responsive interaction—such as empathy, constructive communication, and non-punitive engagement—may enhance relational quality between incarcerated mothers and their children. Additionally, they reported feeling more supported after completing the parenting intervention. Although these changes were statistically significant, the study did not report effect sizes, limiting the ability to assess the strength of program impacts (21).

The *Emotions: Taking Care of Yourself and Your Child When You Go Home* program utilized an in-person interview, the Difficulties in Emotion Regulation Scale (DERS), the Adult Temperament Questionnaire (ATQ), Maternal Emotional Style Questionnaire, Center for Epidemiologic Studies Depression Scale, the Brief Symptom Inventory, and the Self Report Delinquency Scale (22). The findings suggest that mothers who dropped out of the program experienced more depressive symptoms and were more dismissive of their children’s emotions. When the mothers returned home, they reported that the intervention had helped them apply what they had learned—specifically, that the ideas and skills from the class had supported their emotion regulation and helped them respond to their children’s emotions through emotion coaching. Mothers who participated in the intervention were delighted, and early results showed that they had better outcomes than those who did not participate. Specifically, they demonstrated improved emotion regulation, responded with greater support to their children’s emotions, and exhibited fewer criminal behaviors over time, with effects in the moderate-to-large range. By improving mothers’ emotion regulation and supportive responses, this program targets the emotional availability central to secure attachment relationships (22).

The *Parenting Inside Out* program was used in two studies conducted by the same primary author (23, 24). Both studies employed interviews and the Depression, Anxiety, and Stress Scales (DASS-21). However, Collica-Cox and Furst (24) measured self-esteem using the Guttman Self-Esteem Scale, whereas Collica-Cox and Furst (23) measured self-esteem using the Rosenberg Self-Esteem Scale. The findings of the program from Collica-Cox & Furst (24) suggest that the interventions help mothers decrease feelings of depression, anxiety, and parental stress. Additionally, the mothers increased their self-esteem and confidence in their parenting skills. The participants also reported an increase in communication with their children and an improvement in their relationship with their children’s caregivers. Notably, the participants reported improved communication with their family members, staff, and other inmates (24). In contrast, the findings from Collica-Cox and Furst (23) suggest that participants decreased their feelings of depression and increased their parenting knowledge. However, there were no statistically significant changes in other measures, such as anxiety, stress, parental stress, and self-esteem. Participants reported that communication was the most helpful skill they learned, and this skill had helped improve their relationships with family members and their children. These findings highlight the program’s emphasis on improving emotional well-being and relational functioning within and beyond the mother–child relationship. By fostering communication, empathy, and healthier coping strategies, the intervention supports more holistic caregiving practices that strengthen emotional connection and trust between caregivers and children. While statistically significant changes were reported, effect sizes were not reported, and small sample sizes limit generalizability. (23).

The *Parenting While Incarcerated* (PWI) program was reported to have high levels of satisfaction amongst the mothers (25). Participant satisfaction was measured using the *Strengthening Families Program* survey, from which PWI was adapted. The program used group leader process notes to document progress and materials, which topics were covered, and which topics were requested by mothers. The Adult Adolescent Parenting Inventory (AAPI-2) was used to measure parenting attitudes. The findings suggest that the mothers showed lower endorsement for corporal punishment after completing the program than before, although the effects were small in size. This was the only statistically significant finding from the AAPI-2 (25).

Participants in the *Parenting from Prison* program completed the Index of Self-Esteem (ISE), the Adult Adolescent Parenting Inventory (AAPI), a personal history questionnaire, and a follow-up questionnaire after completing the program (26). The results of the program suggest that self-esteem significantly improved, especially among mothers who had frequent visits with their children and were able to write letters to them. Interestingly, empathy scores increased significantly only for mothers who had frequent visits with their children, underscoring how consistent contact and emotional exchange can reinforce the mutual responsiveness central to attachment processes. Participants improved in their understanding of appropriate expectations for behavior, the use of corporal punishment, and parent-child roles. Before the program, many mothers endorsed harsher or more punitive views of discipline, but after completing the intervention, they reported decreased support for corporal punishment and greater endorsement of empathy and positive parenting strategies. Notably, mothers with histories of physical or sexual abuse started with higher prescores, while those without such histories showed the most improvement over time. In addition to changes in parenting attitudes measured by the AAPI, results from the Inmate Self-Evaluation (ISE) and follow-up questionnaires indicated that mothers perceived improvements in their relationships with their children. Specifically, mothers reported feeling more confident in their parenting role, more effective in communicating with their children, and better able to apply newly learned parenting strategies, suggesting enhanced connection and interaction quality following the program. A personal history of the mother being abused and frequently using substances was associated with improved self-esteem at the post-intervention follow-up. Participants who reported frequent alcohol use improved in their roles and other parenting attitudes. In contrast, those who reported frequent drug use showed improvement in expectations of children and parenting attitudes. Although the program yielded statistically significant changes, the authors did not report effect size metrics, which limits the interpretation of the magnitude of these improvements. Overall, the findings suggest that opportunities for ongoing contact, empathy development, and self-reflection can promote meaningful gains in mothers’ confidence and parenting attitudes during incarceration (26).

Across the reviewed studies, interventions varied in their effectiveness depending on their design and focus. Skill-based programs such as *Rebonding and Rebuilding* (21, rated Excellent) and *Parenting from Prison* (26, rated Good) both aimed to shift parenting attitudes, but the methodological quality shaped the strength of the conclusions. Sandifer’s stronger design demonstrated clear reductions in support for corporal punishment and greater empathy among participants, while Thompson & Harm’s program also yielded meaningful improvements in parenting attitudes and self-esteem. However, the more moderate quality of Thompson & Harm’s study—particularly the reliance on limited statistical analyses—means its findings should be interpreted with greater caution. Interaction-based interventions like *PCIT* (20, rated Excellent) and *Parenting From Inside: Making the Mother–Child Connection* (19, rated Excellent) showed the strongest improvements in the quality of mother–child interactions, supporting the idea that opportunities for real-time relational practice amplify program impact. Trauma- and emotion-focused programs, such as *Emotions: Taking Care of Yourself and Your Child When You Go Home* (22, rated Excellent) and *Parenting Inside Out* (23, 24, rated Good), underscored the importance of improving maternal emotion regulation and addressing trauma histories. Shortt’s higher-quality design demonstrated significant improvements in mothers’ emotion regulation and supportive responses to children’s emotions, while the *Parenting Inside Out* studies also showed promising results but were limited by small sample sizes and reliance on self-report measures, constraining their generalizability. Taken together, the most consistent and potent effects came from studies rated “Excellent,” which combined rigorous designs with opportunities for relational practice, while “Good” studies pointed to important statistical outcomes but lacked a comparison group.

### 3.5 Family connections within interventions

In addition to providing education, some interventions specifically assess the effectiveness of their intervention through visitation and caregiver outcomes, demonstrating a holistic approach to improving family connections. For example, the *Parenting from Prison* intervention demonstrated that mothers who maintained contact with their children, either through regular visits or frequent letter exchanges, experienced notable improvements in self-esteem (26). However, mothers who had no contact with their children reported lower self-esteem overall, with those who received no visits remaining in the clinically low range. The intervention also showed that empathy for children’s needs increased significantly among mothers who were visited at least once a month, while it declined for those with less frequent visits. Additionally, mothers who received visits showed improvement in their understanding of parent-child roles and reported changes in their beliefs about corporal punishment. All mothers who received visits reported enhanced frequency and quality of both interactions and written communication with their children (26). These findings emphasize the significance of maintaining family connections to support incarcerated mothers’ overall well-being. From an attachment perspective, maintaining even minimal contact through visits or letters helps sustain emotional availability and a sense of continuity in the caregiver–child relationship, buffering both mothers and children from the psychological effects of prolonged separation.

The *Parenting Inside Out* adapted program has shown varying outcomes regarding caregiver relationships. The findings by Collica-Cox and Furst (24) revealed that several women reported the program helped them improve their relationships with their children’s caregivers. Five participants specifically noted gains in understanding the caregivers’ needs, using more effective communication, and learning to pause and calm down before reacting, which contributed to an increased likelihood of child visitation (24). In contrast, the findings by Collica-Cox & Furst (23) revealed little reported change in caregiver relationships. Two women stated they already had a positive relationship with the caregiver, while one mentioned rarely communicating with them. Three participants felt it was inappropriate to contact caregivers during incarceration. However, they intended to reconnect after release. For four women, caregiver involvement was not applicable due to factors such as children being adults, placed in foster care, or having unknown whereabouts (23). These findings suggest that while *Parenting Inside Out* adapted can foster improved caregiver relationships that support family contact, outcomes may vary depending on individual circumstances and timing.

The *Parenting While Incarcerated* (PWI) intervention incorporates caregiver involvement and visitation components to strengthen family connections (25). To support ongoing communication, mothers were provided with stationery and stamps to write letters or send worksheets to their children and caregivers, sharing the parenting skills they were learning. Some mothers also sent home their program completion certificates to demonstrate their progress. A key focus of the program included improving communication with alternate caregivers and applying skills such as active listening and effective praise during visits and interactions. In collaboration with a community partner agency, the program also facilitated child visitation sessions that allowed for physical contact, enabling mothers to practice their new parenting skills in real-time. Facilitators often attended these visits to offer encouragement and feedback, and they also had the chance to meet the children. While some mothers chose not to have their children visit for various reasons, such as to protect them from the jail environment or because the child was unaware of the incarceration, those who did participate in visitation generally found it to be a meaningful and affirming experience (25). These efforts highlight the intervention’s commitment to preserving and strengthening family bonds, recognizing that meaningful connections with children and caregivers are essential to both the rehabilitation of incarcerated mothers and the well-being of their families.

The *Parenting From Inside: Making the Mother-Child Connection* intervention intentionally included both child visitation and caregiver involvement as key components of its design (19). These elements supported mothers in strengthening their parenting relationships despite the challenges of incarceration. Compared to their pretreatment scores, mothers in the intervention group reported significantly reduced parenting stress related to feelings of competence and the visitation process. They also developed stronger alliances with their children’s caregivers and demonstrated increased communication efforts, including more frequent phone calls, caregiver consultations, and a slight rise in letter writing. The curriculum promoted improved co-parenting relationships and emphasized consistent communication with children through written correspondence. The intervention offered enhanced visitation sessions, supported by an affiliated institutional program and attended by doctoral students, provided additional opportunities for interaction and support among mothers, children, and caregivers (19). These outcomes highlight the importance of integrating both family contact and caregiver collaboration in parenting interventions. It recognizes that maintaining communication and shared parenting roles during incarceration, supports relational stability and co-regulation within the caregiver system.

Taken together, interventions that intentionally integrated visitation and caregiver involvement tended to yield stronger and more durable improvements in family connection. For example, *Parenting From Inside: Making the Mother–Child Connection* (19, rated Excellent) demonstrated the most consistent benefits, including reduced parenting stress, stronger caregiver alliances, and sustained communication through calls and letters. *Parenting While Incarcerated* (25, rated Good) also fostered meaningful child and caregiver contact, but results were limited by small sample size and reliance on self-report. By contrast, *Parenting Inside Out* (23, 24, both rated Good) showed mixed caregiver outcomes—suggesting that individual circumstances, program adaptation, and methodological limitations constrained the findings. *Parenting from Prison* (26, rated Good) reinforced the importance of visitation by showing that mothers with regular contact experienced the greatest improvements in empathy and self-esteem, but the weaker study design limits confidence in these results. Importantly, only one intervention in this category achieved an “Excellent” rating, which may reflect the methodological challenge of using comparison groups to evaluate family connections, given the variability in caregiver involvement and visitation access. Overall, the most robust evidence for fostering family bonds emerges from higher-quality studies that integrate structured visitation and caregiver collaboration. This supports a core concept of attachment theory—the importance of consistent and emotionally responsive interactions to preserve secure mother–child relationships, particularly in the context of incarceration.

## 4 Discussion

This review examined intervention strategies explicitly tailored for incarcerated mothers, with a focus on their effectiveness and connection to family relationships. The findings are needed as incarcerated mothers face distinct challenges, such as separation from children, strained caregiver relationships, and trauma histories, that must be addressed to support family-related outcomes (19). While the interventions varied in structure, content, and delivery, they shared a common goal of strengthening maternal identity and supporting parental skills to improve connections with their children during incarceration and after release. Despite intervention differences, many programs yielded consistent benefits for mothers, including reduced parenting stress, increased parenting knowledge, and improvements in self-esteem or emotional regulation (19–24, 26). These results underscore the importance of designing correctional programming that reflects the unique experiences of justice-involved mothers and acknowledges the relational contexts in which they raise their children.

Parenting interventions for incarcerated mothers can be seen as attempts to restore or preserve these attachment processes despite the disruptive context of imprisonment. By strengthening mothers’ skills in communication, emotional regulation, and caregiving behaviors, interventions reinforce the building blocks of secure attachment. Even in the absence of physical proximity, structured opportunities for letter writing, phone calls, or supervised visits allow mothers to engage in “attachment-preserving behaviors” that mitigate disruptions caused by incarceration. For instance, interventions that integrate child visitation or caregiver collaboration directly enable mothers to demonstrate responsiveness and maintain emotional presence. Conversely, programs that emphasize internal regulation—such as *Emotions: Taking Care of Yourself and Your Child When You Go Home* (22)—equip mothers to manage distress and remain emotionally available when reunification occurs. Thus, interventions may be effective not simply because they transfer parenting knowledge, but because they sustain the relational processes that underlie attachment security, positioning mothers to re-establish bonds with their children during and after incarceration.

Across the reviewed interventions, several shared elements emerged that contributed to improved family-related outcomes among incarcerated mothers. Notably, several programs, such as *Parenting Inside Out*, *Parenting While Incarcerated*, *Parenting from Prison*, and *Parenting From Inside: Making the Mother-Child Connection*, explicitly integrated communication skill-building, emphasizing written correspondence, phone contact, and collaboration with caregivers (19, 23–26). These programs often paired skill development with opportunities for real-time application through child visitation or structured caregiver interaction, which appeared to enhance mothers’ confidence and relational effectiveness. In contrast, programs such as *Parent-Child Interaction Therapy* (PCIT) and *Rebonding and Rebuilding* placed greater emphasis on behavioral skills and parenting attitudes rather than communication, and while they improved parenting practices, they did not directly measure or report changes in maternal communication (20, 21). Similarly, *Emotions: Taking Care of Yourself and Your Child When You Go Home* focused primarily on emotional regulation and internal processes, rather than on external communication with children or caregivers (22). A key difference among the interventions is the degree to which they directly involve children or caregivers, such as through visits, letter writing, or other interactive elements. This suggests that while parenting skills can be taught in isolation, they may be more meaningful when mothers have opportunities to apply them through real-life family contact. At the same time, some interventions showed that even without direct involvement of children or caregivers, improvements in maternal self-awareness, emotional regulation, and parenting attitudes can support future reconnection. These findings suggest there is a shared value of interventions designed specifically for incarcerated mothers, who have unique needs, and emphasize the importance of adapting programs to each mother’s circumstances and level of family support.

At the same time, a skill-focused approach alone may not fully account for the extensive trauma histories that many incarcerated mothers carry. While nearly all of the reviewed interventions emphasized parenting skills, only two programs explicitly integrated trauma-informed components (22–24). This gap is striking given that unresolved trauma, including experiences of victimization, substance use, and psychological distress, directly shapes mothers’ ability to engage in caregiving roles. Without attending to these deeper issues, even well-designed parenting curricula risk overlooking the emotional and relational barriers that incarcerated mothers face. Programs that integrate trauma-informed practices alongside skill development are therefore essential, as they not only build parenting competence but also support mothers in reconstructing a coherent sense of self as caregivers. Ultimately, interventions must adopt a holistic approach that addresses both the relational disruptions of incarceration and the psychological healing needed to sustain meaningful family bonds.

In addition to these shared and unique features, the reviewed interventions also reflected a range of thematic outcomes related to the broader experience of incarceration for mothers. Several studies have highlighted the benefits of general parenting programs, including increased parenting knowledge, improved attitudes toward child discipline, and enhanced relational skills (20, 21, 23–25). Others explored the emotional strain of separation, emphasizing the psychological toll that incarceration takes on mothers who are unable to maintain consistent contact with their children (26). One intervention addressed post-release and reunification challenges, recognizing the need for emotional regulation and preparation as mothers transition back into caregiving roles after release (22). Notably, one intervention also acknowledged intergenerational patterns of incarceration, where maternal justice involvement may reflect a larger cycle of systemic disadvantage and trauma, as seen in Loper and Warren (19). Finally, several interventions, particularly those that integrated trauma-informed practices, recognized how incarceration intersects with mothers’ parenting identities and unresolved trauma, further shaping their ability to reconnect and engage with their children (22, 26, 27). These themes reveal that while parenting interventions often focus on practical skills, many also aim to address the deeper emotional, historical, and systemic contexts that shape mothers’ experiences of parenting during incarceration.

It is crucial to acknowledge the various limitations that arise from different aspects of this review, as well as the corresponding study designs. One limitation involves the review’s exclusion criteria. The interventions analyzed only included mothers who received programming while incarcerated, excluding those who participated in post-release or combined interventions. This review also excluded follow-up studies, programs serving both mothers and fathers, non–English language studies, or those conducted outside the United States and its territories. Additionally, a standard limitation across the included studies was small sample sizes, which were often affected by participant release, facility transfers, or other disruptions within the justice system. Many studies also relied heavily on self-reported outcomes, which may introduce bias due to social desirability or limited recall. Another limitation is the lack of long-term follow-up, which makes it difficult to determine whether the benefits of these programs were sustained after release. Finally, several studies identified limited feasibility of child or caregiver contact due to logistical and emotional barriers such as distance, caregiver willingness, and family stressors; however, research suggests that integrating real-time contact opportunities into interventions may enhance their impact by allowing mothers to apply learned skills and maintain family bonds during incarceration (22).

Despite these limitations, the interventions discussed have made significant contributions to the existing knowledge and understanding of justice-involved mothers and their families. Similarly, these articles have paved the way for further research to add new promising directions. Several implications for practice and policy are also noted. From a practice standpoint, programs should continue to incorporate opportunities for family connection, including caregiver communication and child visitation, as these elements appear to support mothers in maintaining their parenting roles during incarceration. From a policy perspective, supporting incarcerated mothers requires structural changes that make it possible to preserve parent–child bonds while addressing trauma. Correctional institutions should prioritize child-friendly visitation environments and expand opportunities for real-time family contact, such as enhanced phone and video communication. Staff training in trauma-informed and attachment-informed practices can ensure that parenting interventions are delivered with sensitivity to mothers’ lived experiences. Additionally, programs should be designed to bridge incarceration and reentry by maintaining continuity of parenting support after release, reducing the risk of attachment disruptions during reunification. Policies that strengthen caregiver partnerships are also essential, as caregivers act as attachment figures in mothers’ absence and are central to family stability. Although this review did not directly assess mother–baby units (MBUs) or other alternatives to maternal incarceration, a discussion on their inclusion in policy is warranted. MBUs, for instance, represent an attachment-informed approach that allows eligible mothers to remain with their infants during custody, preserving early bonding and promoting maternal rehabilitation. Beyond MBUs, other attachment-informed alternatives also warrant consideration. Community-based residential programs allow mothers to live with their children in noncustodial environments, supporting both rehabilitation and caregiving relationships. Family-centered probation and diversion programs replace incarceration with supervised parenting and counseling, maintaining parent–child contact while addressing risk factors for recidivism. In addition, caregiver-inclusive models strengthen collaboration between mothers and those caring for their children, fostering continuity and shared responsibility in parenting. Together, these approaches offer pathways to preserve caregiver–child bonds while promoting rehabilitation. Though distinct in structure, these approaches share a common theoretical foundation, which stresses maintaining consistent, responsive caregiving relationships to reduce the emotional and developmental harm of separation. Expanding access to such options would align correctional policy with attachment theory’s emphasis on preserving secure relationships, thereby mitigating intergenerational trauma.

While expanding alternative approaches represents an important policy direction, most incarcerated mothers will continue to experience separation from their children within secure facilities. For these women, parenting interventions implemented during incarceration remain the most direct means of maintaining maternal identity, family connection, and skill building. When effectively delivered, such programs can mitigate the emotional toll of separation, enhance coping and emotion regulation, and strengthen the skills needed to sustain family relationships post-release. Continued investment in and adaptation of these interventions can help ensure that mothers have consistent opportunities that promote healthier family reunification. Thus, policy reform should not only expand alternatives but also fund and embed parenting programs within correctional settings as part of a broader commitment to trauma-informed and family-centered rehabilitation.

Future research should explore post-release outcomes, include more diverse and representative samples, and consider the perspectives of caregivers and children to gain a deeper understanding of the broader impact of these interventions. While there is still much to learn, this review highlights the importance of supporting mothers in their parenting roles to strengthen both individual well-being and family cohesion during and after incarceration. Strong family ties have consistently been identified as a protective factor, contributing to reduced recidivism, better mental health outcomes, and more successful reentry for incarcerated individuals, making family-focused interventions a critical area for continued practice and research.

## Data Availability

The original contributions presented in the study are included in the article/supplementary material. Further inquiries can be directed to the corresponding author.
